# Poly (ADP-ribose) polymerase- and cytochrome *c*-mediated apoptosis induces hepatocyte injury in a rat model of hyperammonia-induced hepatic failure

**DOI:** 10.3892/mmr.2015.3281

**Published:** 2015-01-29

**Authors:** GUANMIN GAO, ZUJIANG YU, JINGYA YAN, JINGJING LI, SHEN SHEN, BIN JIA, KELEI GUAN, XIAOJUAN GAO, QUANCHENG KAN

**Affiliations:** 1 Department of Internal Medicine, The First Affiliated Hospital of Zhengzhou University, Zhengzhou, Henan 450052, P.R. China; 2 Department of Infectious Diseases, The First Affiliated Hospital of Zhengzhou University, Zhengzhou, Henan 450052, P.R. China; 3 Department of Pharmacology, The First Affiliated Hospital of Zhengzhou University, Zhengzhou, Henan 450052, P.R. China

**Keywords:** hepatic failure, hepatocyte injury, apoptosis, poly (ADP-ribose) polymerase, cytochrome *c*

## Abstract

Hepatic failure (HF) is caused by several factors, which induce liver cell damage and dysfunction. However, the specific mechanism of HF remains to be fully elucidated. The present study aimed to investigate the underlying cause of hepatocyte injury and liver dysfunction. Liver cells were isolated from healthy female Sprague-Dawley rats, aged between 6 and 8 weeks, weighing ~230 g. The liver cells were cultured in RPMI-1640 medium containing 10% fetal bovine serum. An MTT assay was used to examine the inhibitory rate of liver growth in each group. Flow cytometric analysis was performed to detect liver cells undergoing apoptosis. The protein expression levels of poly (ADP-ribose) polymerase (PARP) and cytochrome *c* (Cyt C) were detected by western blotting. The level of calmodulin-dependent kinase (CaMK) was assessed using an ELISA. The results indicated that the growth inhibitory rate of rat liver cells was significantly increased following treatment with increasing concentrations of NH_4_Cl. The results of flow cytometric analysis demonstrated that the apoptotic rate in the BAPTA-acetoxymethyl ester group was significantly lower compared with the NH_4_Cl group (P<0.05). Treatment with NH_4_Cl increased the protein expression levels of PARP and Cyt C in the liver cells. The mRNA expression of CaMK decreased gradually following treatment with increasing concentrations of NH_4_Cl for 6, 12 and 24 h. The results suggested that hepatocyte injury and liver dysfunction may be caused by inducing apoptosis via the PARP and Cyt C pathways. Additionally, downregulation of CaMK may be associated with the apoptosis observed in hepatocyte injury.

## Introduction

Hepatic failure (HF) is caused by a variety factors, which induce liver cell damage and liver dysfunction. HF is one of the most severe conditions observed in hospital emergencies. Severe HF can cause hepatic encephalopathy, hepatorenal syndrome and hemorrhaging (1,2). The mortality rate of HF is between ~70 and 80% (3,4). High levels of ammonia in the blood may be an important cause of liver abnormalities or failure (5).

It was demonstrated between 2005 and 2011, at The First Affiliated Hospital of Zhengzhou University (Zhengzhou, China), that treating patients with liver failure with ammonia-lowering drugs resulted in remission, reduced patient mortality and improved patient prognosis (6). Our previous study established a chronic hyperammonemia attack rat model and demonstrated that the enzymatic indicators of liver damage were significantly increased (5,7). Pathological investigations demonstrated that hyperammonemia induces liver cell damage, not by inflammation or necrosis, but by cell apoptosis. Excessive hyperammonemia may be important for the regulation of the developmental process of hepatocyte apoptosis and liver damage (7–9).

However, the toxicity of high blood ammonia varies in different cell types (9). At the same concentration of blood ammonia, the growth of McCoy, MDCK, HeLa and BHK cell lines are markedly inhibited, however, the 293, HDF, Vero and PQXB1/2 cell lines are affected less, revealing that the effect of ammonia on cells is cell type-specific. Liver failure caused by elevated blood ammonia can damage neurons or glial cells, leading to hepatic encephalopathy (10). The damage and toxicity of high blood ammonia to liver cells remains to be elucidated. Further investigation into the effect of high blood ammonia on liver cell injury and its underlying mechanism is important in understanding liver failure.

## Materials and methods

### Experimental cells

The present study was approved by the Committee of Ethics in Animal Experiments of the First Affiliated Hospital of Zhengzhou University (Henan, China). Liver cells were collected from six healthy female Sprague-Dawley rats between 6 and 8 weeks old, weighing 230 g. The rats were obtained from Henan Experimental Animal Center (Certificate of Conformity: SCXK (Henan) 2005-0001; Zhengzhou University). The rats were housed in a room with a 12 h light/12 h dark cycle (22±2°C), and were given *ad libitum* access to standard food and water. The rats were treated in compliance with the Institutional Guidelines of Zhengzhou University. BCG-823 gastric cancer cells, 9706 esophageal carcinoma cells and A549 lung cancer cells were donated from Chao Han (Clinical Pharmacology Laboratory).

### Liver cell culture

Six rats were housed in a room with a rotating 12 h light and 12 h dark cycle. The temperature was 22 ± 2°C, and the rats were given standard water and food *ad libitum*. All procedures were approved by the Committee of Ethics in Animal Experiments at Zhengzhou University (Henan, China) and all animals were provided humane care in compliance with the institutional guidelines of Zhengzhou University. Sodium pentobarbital anesthesia is used. The animals were decapitated, submerged in 75% alcohol (Solarbio Science & Technology Co., Ltd., Beijing, China) for 2–3 min and the liver tissues were separated using PBS (Solarbio Science & Technology Co., Ltd.) at 4°C. Following the removal of capsule and fabric composition, the livers were cut into small sections (1 mm^3^) using a one-sided blade and cleaned twice with PBS at 4°C to remove fragments from the cutting process. The small tissue sections were added to 15 ml collagenase IV in 0.05% Dulbecco’s modified Eagle’s medium (DMEM; Solarbio Science & Technology Co., Ltd.) and digested for 30 min in a 37°C incubator. Clockwise agitation was performed every 5–10 min and, following digestion, the tissue sections were pipetted, filtered using a 120 mesh strainer (Solarbio Science & Technology Co., Ltd.) and cleaned with 5 ml PBS. The cell suspension (~20 ml) was collected separately into two micro-centrifuge tubes (Solarbio Science & Technology Co., Ltd.), prior to centrifugation for 2 min at 40 × g three times. Percoll separation liquid (5 ml; Solarbio Science & Technology Co., Ltd.) and the cell suspension were added to every sample (5:3 ratio), and centrifuged for 10 min at 118–161 × g. The cell suspension (5×10^5^ cells/ml) required careful addition onto the surface of the separation liquid using a pipette. The purified liver cell suspension (2–3 ml) was pipetted from the bottom of the tube into 5 ml DMEM and stained with 0.4% trypan blue (Solarbio Science & Technology Co., Ltd.). For subsequent experiments, the required survival rate of cells was >90%.

The rat liver cells, which were anchorage-dependent, were cultured in RPMI-1640 medium (Solarbio Science & Technology Co., Ltd.) containing 10% fetal bovine serum (FBS) (Solarbio Science & Technology Co., Ltd.) in an 37°C incubator (5% CO_2_ and 80% humidity). The cells were passaged every 2–3 days once confluence of 80% had been reached. When the cultured cells grew against the wall of flask, the logarithmic growth phase cells were collected for further investigation.

### Determination of modeling concentration using an MTT colorimetric assay

The rat liver cells (10^5^ cells/ml) were seeded into 96-well culture plates with RPMI-1640 medium supplemented with 10% FBS (100 *μ*l/well). Following growth to the appropriate density (5×10^5^ cells/ml), NH_4_Cl solution was added to the cells at a final concentration of 5, 10, 20, 40, 80 or 100 mM, and the cells were divided into a cell culture free group and a normal control group (four wells per group). The BCG-823 gastric cancer cells, 9706 esophageal carcinoma cells and A549 lung cancer cells were cultured in NH_4_Cl solution at identical concentrations (5×10^5^cells/ml). After 24 h, 20 *μ*L MTT (5 mg/ml PBS) was added to the wells. Following incubation for 4 h at 37°C, the supernatant was discarded. Dimethyl sulfoxide (200 *μ*l) was added to each well and mixed thoroughly in the dark. Finally, the optical density (OD) was measured at 490 nm using an enzymatic analyzer (NanoDrop 2000; Thermo Fisher Scientific, Waltham, MA, USA). Inhibition of cell proliferation was calculated using the following formula: Inhibition ratio = 1 − OD mean value of trial group / control group) × 100%. The half maximal inhibitory concentration (IC_50_) was calculated using IC_50_ software (Calcusyn 2.0; Biosoft, Cambridge, UK).

The rat liver cells were divided into six groups (four wells per group) in 96-well culture plates (Suzhou ConRem Biomedical Technology Co., Ltd., Suzhou, China). A control group remained untreated and the cells in the NH_4_Cl treatment group were cultured in 2.5, 5, 10, 20, 40 or 50 mm NH_4_Cl (Solarbio Science & Technology Co., Ltd.). In the EGTA group, 2 mM EGTA (Solarbio Science & Technology Co., Ltd.) was added at the same time as the NH_4_Cl. Another three groups were treated with 0.1, 0.01 or 0.001 *μ*mol/l BAPTA-acetoxymethyl ester (BAPTA-AM; Solarbio Science & Technology Co., Ltd.) 40–60 min at 37°C prior to NH_4_Cl treatment. Continue to develop for 6 h, the MTT assay was then performed, as described above.

### Determination of cell survival and cell growth using an MTT colorimetric assay

The rat liver cells (10^5^ cells/ml) were seeded into 96-well culture plates (100 *μ*l/well) with RPMI-1640 medium supplemented with 10% FBS (100 *μ*l/well). Following growth to the appropriate density (5×10^5^ cells/ml), NH_4_Cl solution was added at a final concentration of 2.5, 5, 10, 20, 40 or 50 mM and the cells were divided into a cell culture free group and a control group (PBS; four wells per group). The cells were cultured for 24 and 48 h, respectively, and the medium was replaced every 6 h. In the BAPATA-AM groups, BAPATA-AM was added 40–60 min prior to treatment with NH_4_Cl at a final concentration of 0.01 *μ*M. By contrast, in the EGTA groups, the EGTA and NH_4_Cl were added at the same time at a final concentration of 2 mM. The other treatments performed were as described above. The inhibition of cell proliferation was calculated using the following formula: Inhibition ratio = 1 − mean OD of trial group / control group) × 100%.

### Determination of cell apoptosis using flow cytometry

The rat liver cells (2 ml; 1×10^5^/ml) were uniformly seeded into 6-well culture plates. Following 18–24 h culture, NH_4_Cl solution was added at concentrations of 0, 1, 5, 10 or 20 mM, and the cells were cultured for a further 24 h. The liver cells were collected by trypsin enzyme-digesting (Solarbio Science & Technology Co., Ltd.) without EDTA and the cell suspension samples (5×10^5^ cells) were obtained for detection. Following washing twice with PBS, the rat liver cells were resuspended (5×10^5^ cells/ml) in 2 ml binding buffer (Solarbio Science & Technology Co., Ltd.) and incubated with fluorescein isothiocyanate at room temperature for 20 min in the dark, prior to being centrifuged for 5 min at 118 × g. The cells were resuspended in 2 ml binding buffer and 2 *μ*l propidium iodide (500 mg/ml; Solarbio Science & Technology Co., Ltd.) was added for 20 min in the dark, prior to being centrifuged for 5 min at 118 × g. The collected cells were resuspended in 2 ml PBS. The quantity of apoptotic cells was measured by flow cytometry (XL-MCL; BD Biosciences, Franklin Lakes, NJ, USA) immediately following staining (excitation wavelength, 488 nm; emission wavelength, 530 nm).

Rat liver cells (2 ml; 1×10^5^ cells/ml) were uniformly seeded into 6-well culture plates. Following 18–24 h culture at 37°C, NH_4_Cl solution was added at a final concentration of 0, 2, 10, 20 or 40 mM and the cells were cultured for a further 6 h. In the BAPATA-AM groups, BAPATA-AM was added for 40–60 min at 37°C prior to treatment with NH_4_Cl at a final concentration of 0.01 *μ*M. Flow cytometry was then performed, as described above.

### Determination of mitochondrial permeability transition pore (MPTP)

Rat liver cells (2 ml; 1×10^5^ cells/ml) were uniformly seeded into 6-well culture plates. Following 18–24 h culture, NH_4_Cl solution was added at a final concentration of 0 or 10 mM. In the BAPATA-AM groups, BAPATA-AM was added 40–60 min prior to treatment with NH_4_Cl at a final concentration of 0.01 *μ*M and cultured for a further 24 h with the medium changed every 6 h. The liver cells were collected using trypsin enzyme-digesting without EDTA, centrifuged for 5 min at 118 × g and the supernatant was discarded. Following a single wash with PBS, the cell samples were washed twice with 37°C preheated cleaning buffer, containing stain fluid (Solarbio Science & Technology Co., Ltd.) and neutralization liquid (1:100; GenMed Scientifics Inc., Shanghai, China) for 20 min at 37°C in the dark. Following washing, MPTP was measured by flow cytometry (XL-MCL), which was analyzed once every 10 min with at least 10,000 cells assessed at each time point (excitation wavelength, 488 nm; emission wavelength, 530 nm).

### Determination of the expression of target proteins by western blot analysis

#### Cell protein extraction

The rat liver cells (2 ml; 1×10^5^ cells/ml) were uniformly seeded into 6-well culture plates. Following 18–24 h culture, NH_4_Cl solution was added at a final concentration of 0, 1, 5, 10 and 20 mmol/l and cultured for a further 24, 48 or 72 h. The culture media was discarded and the cell samples were washed gently with pre-cooled PBS (4°C; 0.01 M; pH 7.2–7.3) three times. Following washing, 200 *μ*l phenylmethanesulfonyl fluoride (PMSF)-based lysis solution containing 50 mmol/l Tris-HCl, (pH8.0), 150 mmol/l NaCl, 1% TritonX-100 and 100 *μ*g/ml PMSF, was added for 30 min on ice. The cells were transferred rapidly into microcentrifuge tubes to centrifuge at 14,000 rpm for 8 min at 4°C. The supernatant was collected and stored at −20°C.

#### Determination of protein concentrations

According to the number of protein standards and the number of samples, bicinchoninic acid (BCA) working fluid was prepared with reagent A and reagent B (50:1; Solarbio Science & Technology Co., Ltd.). A lyophilized standard panel was firstly diluted into 10 mg/ml stock-solution containing 1 ml NaCl (0.9%), and further diluted to 25–2,000 *μ*g/ml for the standard curve. Standard marker (25 *μ*l; Solarbio Science & Technology Co., Ltd.) and the sample were added into 96-well plates with 200 *μ*l BCA working fluid. Following mixing, the 96-well plate was covered and incubated for 30 min at 37°C and subsequently cooled to room temperature. The protein concentration was calculated using a Nanodrop 2000.

#### Determination of the protein expression levels of poly (ADP-ribose) polymerase (PARP) and cytochrome c (Cyt C)

Protein samples (30 *μ*g) were adjusted to an identical volume and 4 *μ*l 6X loading buffer was added prior to samples being heated for denaturation (95°C). The proteins were separated on 12% sodium dodecyl sulphate polyacrylamide gel electrophoresis gels, transferred onto a polyvinylidene difluoride membrane and the membrane was blocked with blocking buffer (membranes and buffer from Solarbio Science & Technology Co., Ltd.) at 4°C for 1 h. The membrane was incubated with primary antibodies against Cyt C (1:3,000; Bioss, Shanghai, China) rat monoclonal PARP (1:3,000; sc-71851, Santa Cruz Biotechnology, Dallas, TX, USA) overnight at 4°C and then a horseradish peroxidase enzyme-labeled secondary antibody (1:1,000) at 37℃ for 1 h. The protein expression levels were determined using an Odyssey CLX system (LI-COR Biosciences, Lincoln, NE, USA).

### Determination of the mRNA expression levels of calmodulin (CaM) and CaM-dependent kinase (CaMK)II by reverse transcription quantitative polymerase chain reaction (RT-qPCR)

#### RNA extraction

Rat liver cells (2 ml; 1×10^5^ cells/ml) were uniformly seeded into 6-well culture plates. Following 18–24 h culture, NH_4_Cl solution was added at a final concentration of 0, 1, 5, 10 or 20 mmol/l and further cultured for 12, 24 or 48 h. The supernatant was discarded. TRIzol reagent (1 ml; Invitrogen Life Technologies, Carlsbad, CA, USA) was added and the cells were incubated for 5 min on ice, prior to pipetting. The cells were transferred to Eppendorf tubes, 0.2 ml chloroform was added and shaken rapidly for 15 min, incubated for 5 min at room temperature and centrifuged for 10 min at 161 × g at 4°C. The upper colorless aqueous phase, which included the RNA, occupied ~50% of the homogenate. The supernatant (400 *μ*l) was carefully transferred to a separate Eppendorf tube and 0.5 ml isopropyl alcohol was added for 10 min at room temperature, prior to being centrifuged for 10 min at 161 × g at 4°C. Small milky sediments, containing RNA, were collected at the bottom of the tube. The supernatant was discarded and the RNA was washed with precooled 75% ethanol, centrifuged for 5 min at 161 × g at 4°C and the supernatant was discarded. The RNA was dried in a sterile environment using a vacuum and subsequently dissolved in 20 *μ*l diethylpyrocarbonate (DEPC) to determine the purity and concentration of the RNA by Nanodrop. The samples were stored at −80°C.

#### RT of RNA to cDNA

The extracted RNA was dissolved in 11 *μ*l DEPC water. Oligo (dT; 1 *μ*l) and primer (0.5 *μ*g/*μ*l; 18 *μ*l; Takara Bio, Inc., Otsu, Japan) were added and mixed gently, prior to centrifugation for 3–5 sec and heated at 70°C for 5 min in a water bath. Following cooling the samples on ice and short centrifugation, 4 *μ*l 5X reaction buffer, 1 *μ*l Ribolock™ ribonuclease inhibitor (20 *μ*g/*μ*l) and 2 *μ*l 10 mM dNTP mix were added (Takara Bio, Inc.), mixed gently and centrifuged for 3–5 sec. The samples were then amplified by PCR for 5 min at 37°C. RevertAid™ M-MuLV Reverse Transcriptase (1 *μ*L; 200 *μ*g/*μ*l; Takara Bio, Inc.) was added and PCR was performed for 60 min at 42°C and 10 min at 70°C. Finally, the reaction products were collected and cooled on ice.

#### qPCR

The qPCR was performed using a SYBR^®^ Premix Ex Taq™ II test kit (Takara Bio Inc.). The cDNA (2 *μ*l; 100 ng/*μ*l), 2X SYBR^®^ Premix Ex Tap™ II (10 *μ*l) and 0.8 *μ*l of each fluorescence quantitative primer (CaM1, forward 5′-TCAACATCTCCTCTACCAACCA-3′ and reverse 5′-TACTCCCTGTCTTTCTGGCATT-′3, and CaMKII, forward 5′-ACCAGCTCTTCGAGGAATTG-3′ and reverse 5′-GTGACCAGGTCGAAGATCAG-3′) and ROX (0.4 *μ*l; Takara Bio, Inc.) were added in a reaction mixture. Reference dye (50X; Invitrogen Life Technologies) and 6 *μ*l distilled H_2_O were added to the reaction at a total volume of 20 *μ*l and qPCR amplification was performed using a StepOnePlus™ Real-Time PCR system (Beckman Coulter, Brea, CA, USA). The following PCR program was used: 95°C for 30 sec denaturation and 40 cycles of 95°C for 5 sec and 64°C for 34 sec. The comparative threshold (Ct) value was obtained from the fluorescent qPCR: Rate F (following treatment / prior to treatment) = 2 −[Ct2 treatment − Ct2 control) − (Ct1 treatment − Ct1 control], which indicated changes in the mRNA expression following and prior to treatment. Rate F refers to the ratio of Ct before (Ct1) and after (Ct2) treatment.

#### Determination of CaMK II by ELISA

Rat liver cells (2 ml; 1×10^5^ cells/ml) were uniformly seeded into 6-well culture plates. Following 18–24 h culture, NH_4_Cl solution was added at a final concentration of 0, 1, 5, 10 or 20 mmol/l and further cultured for 24, 48 or 72 h. The culture media was discarded and the cells were transferred to 10 ml centrifuge tubes containing 0.25 pancreatin (Solarbio Science & Technology Co., Ltd.) without EDTA, prior to being centrifuged for 10 min at 37°C at 800 rpm, and the supernatant was discarded. The precipitate was transferred into 1.5 ml microcentrifuge tubes, resuspended in PBS, centrifuged for 10 min at 10,000 rpm and the supernatant was collected and stored at −20 or 4°C. The CaMKII value was determined as follows. The Human CAMKII ELISA kit (R&D Systems, Inc., Minneapolis, MN, USA) was incubated at room temperature for 15–30 min and 50 *μ*l standard solution (0.1 g BCA added to 0.2 g KH_2_PO_4_, 2.9 g Na_2_HPO_4_.12H_2_O, 8.0 g KCl, 0.5 ml 0.05% Tween-20, 1,000 ml distilled water; Solarbio Science & Technology Co., Ltd.) was added into each well of the ELISA plate, according to the sequence of the standard solution. The cell supernatant (50 *μ*l), which was diluted five-fold, was added to each well three times. The enzyme labelled solution (100 *μ*l) was added, joint sealant, sealers and was incubated for 1 h at 37°C. Maintaining the same indoor temperature and relative humidity, the ELISA plate was washed between three and five times with washing fluids (0.2 g KH_2_PO_4_, 2.9 g Na_2_HPO_4_.12H_2_O, 8.0g NaCl, 0.2 g KCl, 0.5 ml 0.05% Tween-20, made up to 1 L with distilled water) diluted 100-fold and patted dry with blotting paper. Chromogenic agent A and B (50 *μ*l) were added and the reaction was performed in the dark for 15 min. Following the incubation, termination solution (50 *μ*l; Solarbio Science & Technology Co., Ltd.) was added to each well and rested for 5 min at 37°C in order to terminate the reaction. The OD value was measured in an enzymatic analyzer at 450 nm. A standard curve was determined and the corresponding concentration was obtained using this curve.

#### Statistical processing

The experimental data were analyzed using SPSS 17.0 software (SPSS, Inc., Chicago, IL, USA) and data are expressed as the mean ± standard deviation. The mean differences are expressed as 95% confidence intervals. The levels of ammonia in the blood and the relative expression of SPP1 were determined using a Kruskal-Wallis test and differences between groups were determined using a Nemenyi test. Other comparisons were performed using a single factor analysis of variance and differences between groups were determined by the least significant difference test. P<0.05 was considered to indicate a statistically significant difference.

## Results

### Determination of the optimal concentration of NH_4_Cl

As shown in Table I, treatment with identical concentrations of NH_4_Cl led to a different growth inhibitory rates in different cell lines, with the most significant effect was on the liver cells. Treatment with 100 mM NH_4_Cl increased the inhibitory rate to 89.52±0.47%. The IC_50_ of rat liver cells was calculated to be 41.6 mM.

### Determination of optimal drug concentration of the control

High concentrations of NH_4_Cl and EGTA solution and different concentrations of BAPTA-AM were used to treat the cells. The growth inhibitory effect was determined in all cells following a short treatment duration. As shown in Table II, following 6 h treatment, the inhibitory rate increased as the concentration of NH_4_Cl increased in each drug groups. No significant difference were observed between the EGTA and NH_4_Cl groups after 6 h treatment at identical drug concentrations. However, the growth rate of the BAPTA-AM group was significantly inhibited at concentrations >40 mM (P<0.05), particularly in the 0.01 *μ*M group, compared with the NH_4_Cl group.

The results demonstrated that the optimal concentration range of NH_4_Cl for short duration treatments was 0, 2.5, 10, 20 and 40 mM, and for long duration treatments was 0, 1, 5, 10 and 20 mM. The optimal BAPTA-AM concentration was 0.01 *μ*M.

### Detection of cell apoptosis by flow cytometry

**Figure 1:**
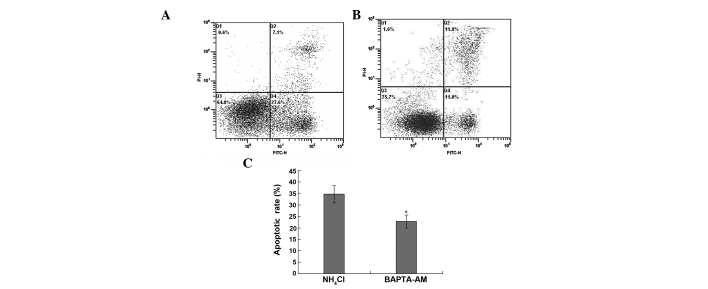
Detection of apoptotic cells following 40 mM NH_4_Cl treatment for 6 h in the NH_4_Cl and BAPTA-AM groups. (A) NH_4_Cl group. (B) BAPTA-AM group. (C) Quantification of flow cytometry data. Data are expressed as the mean ±standard deviation. PI, propidium iodide; FITC, fluorescein isothiocyanate.

In the high NH_4_Cl concentration group and the BAPTA-AM group, the growth of cells was inhibited following a short treatment duration (Fig. 1). When treated with NH_4_Cl for 6 h at a final concentration of 0, 2, 10, 20 or 40 mM, the apoptotic rate in the BAPTA-AM group was significantly lower compared with the NH_4_Cl group (Fig. 1).

### Protein expression of PARP increases in the NH_4_Cl treated group

**Figure 2:**
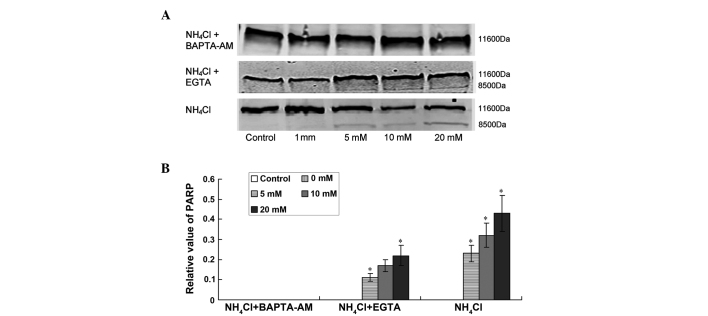
Protein expression of PARP following treatment with different concentrations of NH_4_Cl in rat liver cells with or without pretreatment with EGTA or BAPTA-AM. (A) Western blot analysis for the protein expression of PRAP. (B) Statistical analysis of the expression of PRAP (^*^P<0.05, compared with the former concentration). PARP, poly (ADP-ribose) polymerase.

As shown in Fig. 2, the expression of PARP changed following treatment for 72 h in the NH_4_Cl group and the EGTA + NH_4_Cl group. The protein expression of PARP increased significantly following increasing of the final NH_4_Cl concentration to 5.0, 10.0 and 20.0 mM (Fig. 2; P<0.05). However, no change was detected in the BAPTA-AM group (P>0.05).

### NH_4_Cl induces the protein expression of Cyt C

**Figure 3:**
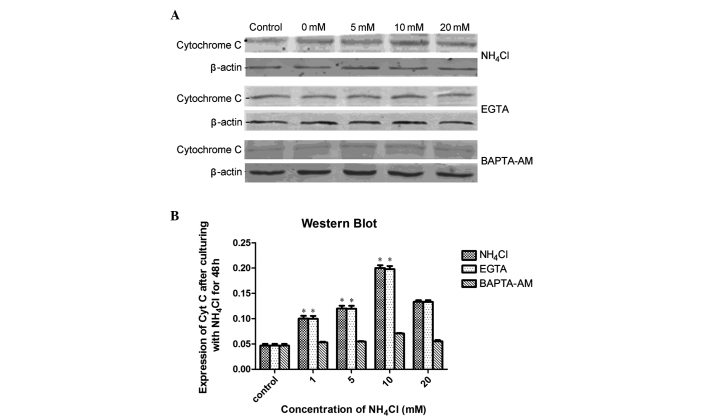
Detection of the expression of Cyt C following treatment with different concentrations of NH_4_Cl in rat liver cells with or without pretreatment with EGTA or BAPTA-AM by western blot analysis. (A) Western blot analysis of the expression of Cyt C. (B) Statistical analysis of the expression of Cyt C (^*^P<0.05, compared with the former concentration). Cyt C, cytochrome *c*; PARP, poly (ADP-ribose) polymerase.

As shown in Fig. 3, the expression of Cyt C increased as the concentration of NH_4_Cl increased, to 10 mM in a dose-dependent manner. However, 20 mM treatment reduced the expression of Cyt C. No significant difference was revealed between the EGTA group and the NH_4_Cl group, which also increased dose-dependently, and the expression decreased following treatment with 20 mM NH_4_Cl. The expression of Cyt C remained unchanged in the BAPTA-AM group.

### NH_4_Cl decreases the expression of CaMK

The rat liver cells were treated with different concentrations of NH_4_Cl for 6, 12 and 24 h. No significant difference was observed in the mRNA expression of CaM, remained unchanged as the treatment concentration and duration increased.

**Figure 4:**
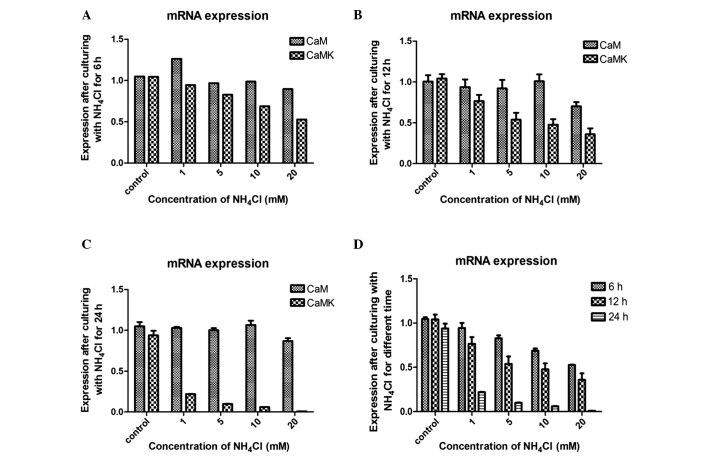
Differences in the mRNA expression following treatment with NH^4^Cl for 6, 12 and 24 h. No statistically significant differences were observed in the expression of CaM at 6 h (P=0.0952), 12 h (P=0.0658) or 24 h (P=0.1058) using an independent t-test. A statistically significant difference was observed between the expression levels of CaMK at all time points (^*^P<0.05). (A) Treatment for 6 h. (B) Treatment for 12 h. (C) Treatment for 24 h. (D) Difference in the mRNA expression levels following treatment with NH_4_Cl for different durations. CaM, calmodulin; CaMK, calmodulin-dependent kinase.

As shown in Fig. 4A, the mRNA expression of CaMK decreased gradually as the concentration of NH_4_Cl increased following 6 h treatment. The results after 12 and 24 h treatment were similar, as shown in Fig. 4B and C. It was demonstrated that the mRNA expression of CaMK decreased as the duration increased following treatment with identical concentrations of NH_4_Cl (Fig. 4D).

## Discussion

In an acute liver failure model, blood ammonia is not only the result of liver damage, but is also the cause of liver damage (11,12). The apoptosis of liver cells may be the underlying mechanism of liver failure. When blood ammonia induce liver damage, cell apoptosis is one of the significant changes to occur and is also the first change observed following blood ammonia treatment in an acute liver failure model (13–15). Cell apoptosis may be the important mechanism and the initial factor causing blood ammonia-induced liver damage. The present study aimed to investigate how this cell apoptosis is induced.

The results demonstrated that liver cells were damaged following ammonia treatment under identical conditions. The growth of cells was markedly inhibited, which was concentration- and time-dependent. Flow cytometric analysis revealed that the cell apoptosis ratio increased with increasing concentrations of NH_4_Cl. This demonstrated that high blood ammonia levels may lead to liver cell damage and apoptosis. At a shorter treatment duration and low concentration of NH_4_Cl, cell apoptosis was the predominant effect and, as the concentration of NH_4_Cl increased and the treatment duration lengthened, the number of apoptotic cells increased significantly. These results demonstrated that cell apoptosis was the predominant effect in the early injury of liver cells, induced by high levels of blood ammonia.

The MTT results, with the exception of the 6 h high concentration treatment or 24 and 48 h low concentration treatment, revealed that the inhibition on cell growth following BAPTA-AM pretreatment was alleviated significantly compared with the other two groups. The rate of cell apoptosis in the BAPTA-AM group was between 30 and 40% lower compared with the NH_4_Cl group following 6 h treatment, however, no inhibition was observed in any of the EGTA treatment groups. This indicated that the liver cell damage by ammonia resulted from the increasing intracyto-plasmic calcium ion concentration and not from changes in the extracellular calcium ion concentration. Intracellular calcium ion chelating agent, BAPTA-AM, can inhibit the occurrence of cell apoptosis by decreasing the intracellular calcium ion concentration, which may be required for liver cell protection from ammonia poisoning or to delay the progress of liver failure by inhibiting cell apoptosis.

MPTP is a nonspecific channel localized in the inner and outer mitochondrial membrane, and is dependent on calcium ions (16). When cell apoptosis or necrosis are induced, mitochondrial content is released into the cytoplasm by MPTP (17,18). The fine structure of the MPTP remains to be elucidated, however, it includes adenosine inversion, the voltage dependent anion channel, peripheral-type benzodiazepine receptor, cancer protein, hexokinase and creatine kinase. Factors, including excessive calcium ions in the cells, mitochondrial glutathione oxidation and increasing reactive oxygen species, cause the MPTP to open, resulting in a change in mitochondrial permeability, releasing Cyt C and resulting in the loss of mitochondrial membrane potential (19). The increased concentration of intracellular calcium ions caused by NH_4_Cl leads to opening of the MPTP and activation of cell apoptosis resulting from the mitochondrial pathway. According to the shift in the fluorescence peak to the left following fluorescent quenching, cell fluorescence in the 10 mM NH_4_Cl group decreased compared with the control group as the treatment duration prolonged and the peak value shifted to the left. However, following BAPTA-AM preprocessing, the degree and speed of the shift to the left were reduced, which demonstrated that BAPTA-AM inhibited the opening of the MPTP by increasing calcium ions. This indicated that NH_4_Cl caused an increase in intracellular calcium ions and led to the opening of the MPTP and apoptosis. However, treatment with BAPTA-AM reduced the opening of MPTP through its calcium chelating effect to reduce the rate of cell apoptosis.

The opening of the MPTP causes an increase in Cyt C release (20). Cyt C is a necessary molecule for cell survival and also a promoter molecule for cell death (21). Cyt C enters the cytoplasm through an open MPTP, induces apoptosis proteinase activating factor and activates homocysteine, this induces a caspase cascade reaction and activates caspase 3 to hydrolyze the substrate proteins, including cleaved PARP, DNA and steroid hormone response element protein 1 and 2, eventually leading to the occurrence of apoptosis (22). High concentrations of ammonia alters the mitochondrial permeability of star-shaped glial cells cultured *in vitro*, causing severe apoptosis or necrosis of the glial cells, which is controlled by the concentration of calcium ions (23,24). Cyt C is important in electron transfer for biological oxidation and can form a respiratory chain with other oxidases on the mitochondrial cristae. A lack of Cyt C in the mitochondrial respiratory chain may cause electron transfer problems, the production of reactive oxygen species, cell damage and can lead to cell apoptosis, and oxidative damage. This occurs in the mitochondria and is part of the mitochondrial apoptotic pathway (25). Therefore, it is important to investigate the expression of Cyt C to understand the mechanisms underlying cell apoptosis.

PARP is an important substrate cleaved by caspase 3 (26) and is predominantly associated with DNA repair, ensuring complete transcription and expression of genes. Activated caspase 3 cleaves PARP into two fragments, preventing the interaction with DNA. Impaired PARP function increases the Ca^2+^/Mg^2+^-dependent endonuclease activation, damages nucleosome structure and increases the occurrence of apoptosis. Specific inhibitors of caspase 3 inhibit the occurrence of apoptosis and the degradation of PARP can activate the caspase cascade reaction (27).

In the present study, key proteins in the mitochondrial apoptotic pathways, Cyt C and PARP, were assessed by western blotting. Following treatment with NH_4_Cl for 72 h, PARP was partly hydrolyzed in all the cells treated with 5, 10 and 20 mM and the expression of Cyt C increased. The results demonstrated that the high blood ammonia caused the release of Cyt C from the mitochondria, activated the cascade reaction, increased the degradation of PARP and eventually induced the rate of apoptosis. However, following pretreatment with BAPTA-AM, no significant difference in the expression levels of Cyt C and PARP were observed, suggesting that BAPTA-AM effectively chelated the intracellular calcium, preventing the mitochondria damage caused by calcium overload and released apoptotic factors to protect the liver cells. Notably, that the expression of Cyt C in the 20 mM group decreased compared with the 10 mM group, which may be caused by the rapid increase in calcium ions resulting from the treatment with excessive concentrations of NH_4_Cl. This may also induce necrosis of certain cells causing a reduction in the number of cells undergoing apoptosis.

CaM is a calcium-dependent activated calcium-binding protein. When combined with a calcium ion, CaM undergoes changes in configuration, activates certain enzymes and regulates cell activity. CaMK is a serine/threonine protein kinase and its activity is adjusted by the calcium/calmodulin complexes. Previous studies have revealed that the activation of Ca^2+^-CaM-CaMK may be involved in the regulation of cell apoptosis (28,29). The results from fluorescence qPCR and ELISA demonstrated no significant increase in the expression levels of the CaM and CaMK in the Ca^2+^-CaM-CaMK signaling pathway, which indicated that the apoptotic pathway was not activated. However, the mRNA expression of CaMK decreased as the duration and concentration of treatment increased, which may be associated with the effect of reduced adenosine triphosphate (ATP) synthesis following mitochondrial damage. However, the concentration dependency may be associated with high blood ammonia, which affects CaMK and cell proliferation.

Importantly, early treatment with NH_4_Cl may cause an increase in the levels of calcium ions in liver cells, leading to the opening of the MPTP, activation of the mitochondrial apoptosis pathway, release of Cyt C and degradation of PARP induced by an activated caspase cascade reaction (29–31). In this process, the mitochondria are damaged and the production of ATP, protein synthesis, cell proliferation and other activities may be affected. Therefore, increased levels of ammonia in the blood may be an important cause of further damage to residual liver cells following liver failure. It is important to investigate the underlying mechanism of liver cell damage by blood ammonia to further understand the pathogenesis of liver failure and to identify novel therapeutic targets and techniques.

## Figures and Tables

**Table I:** Growth inhibitory rate of different types of cells following treatment with NH_4_Cl for 24 h.

NH_4_Cl concentration (mM)	Growth inhibitory rate (mean % ±standard deviation)			
	Rat liver	BCG-823	9706	A549
100	89.52±0.47^a^	17.81±1.14^b^	16.60±1.52^b^	14.24±0.64^b^
80	80.26±0.60^a^	13.97±0.87^b^	11.99±2.38^b^	11.47±1.43^b^
40	33.55±1.52^a^	11.86±2.39^b^	8.05±2.58^b^	8.74±0.85^b^
20	16.71±1.25^a^	5.20±2.16^b^	3.69±1.48^b^	7.75±0.83^b^
10	12.91±0.71^a^	2.61±0.59^b^	2.08±1.18^b^	6.77±0.67^b^
5	9.81±0.84^a^	1.02±0.50^b^	1.44±1.60^b^	4.65±0.29^b^

Two-way analysis of variance was used. Statistically significant differences were observed between the two groups (^a^P<0.01, compared with the untreated cells). A least significant difference test was used, and the difference between the two groups was statistically significant (^b^P<0.05, compared with the untreated cells).

**Table II:** Growth inhibitory rate (%) of different groups of rat liver cells following treatment with NH_4_Cl for 6 h.

NH_4_Cl concentration (mM)	BAPTA-AM				
	NH_4_Cl	EGTA	0.1 *μ*M	0.01 *μ*M	0.001 *μ*M
100	49.25±1.71	46.50±2.38	29.32±1.45^a^	26.02±1.32^a^	28.44±1.77^a^
80	36.93±1.14	35.00±1.09	25.26±1.92^a^	23.71±1.88^a^	25.02±1.63^a^
60	27.96±1.30	26.02±1.32	22.93±2.13^b^	20.43±0.83^a^	22.45±1.46^b^
40	23.29±2.90	23.71±1.88	16.67±2.12^b^	15.28±2.20^b^	16.87±1.97^b^
20	14.04±2.34	13.63±1.50	12.53±1.98	11.09±2.01	12.33±2.78
10	9.23±1.54	9.07±2.00	8.23±1.12	7.40±0.93	8.19±0.97

Two-way analysis of variance was used and statistically significant differences were observed between the BAPTA-AM groups Data are expressed as the mean percentage ±standard deviation. (^a^P<0.01 and ^b^P<0.05, compared with untreated rat liver cells).

## References

[1] Jones EA, Mullen KD (2012). Theories of the pathogenesis of hepatic encephalopothy. Clin Liver Dis.

[2] Zamora Nava LE, Aguirre Valadez J, Chávez-Tapia NC, Torre A (2014). Acute-on-chronic liver failure: a review. Ther Clin Risk Manag.

[3] Bernal W, Wendon J (2013). Acute liver failure. N Engl J Med.

[4] Yang Q, Shi Y, Yang Y, Chen Z (2013). Deactivation and aoptosis of hepatic macrophages are involved in the development of concanavalin A-induced acute liver failure. Mol Med Rep.

[5] Jia B, Yu ZJ, Duan ZF (2014). Hyperammonaemia induces hepatic injury with alteration of gene expression profiles. Liver Int.

[6] Liu CP, Yu ZJ (2011). Study on L-Ornithine-L-Aspartate in the treatment of acute-on-chronic liver failure. Zhonghua Gan Zang Bing Za Zhi.

[7] Yu ZJ, Sun R, Liu XR (2013). Hyperammonemia-induced hepatic injury in rats: characterization of a ne w animal model. Zhonghua Gan Zang Bing Za Zhi Zhonghua Gan Zang Bing Za Zhi.

[8] Lao MS, Toth D (1997). Effects of ammonium and lactate on growth and metabolism of a recombinant Chinese hamster ovary cell culture. Biotechnol Prog.

[9] Helgeland K (1981). NH4Cl and protein metabolism in human gingival fibroblasts. Scand J Dent Res.

[10] Hassell T, Gleave S, Butler M (1991). Growth inhibition in animal cell culture. The effect of lactate and ammonia. Appl Biochem Biotechnol.

[11] Kasahara I, Saitoh K, Nakamura K (2000). Apoptosis in acute hepatic failure: histopathological study of human liver tissue using the tunel method and immunohistochemistry. J Med Dent Sci.

[12] Malhi H, Guicciardi ME, Gores GJ (2010). Hepatocyte death: a clear and present danger. Physiol Rev.

[13] Chen Z, Chen Y, Chen J, Shen C (1992). Effects of ammonium and lactate on hybridoma cell growth and metabolism. Chin J Biotechnol.

[14] Doyle C, Butler M (1990). The effect of pH on the toxicity of ammonia to a murine hybridoma. J Biotechnol.

[15] Cruz HJ, Freitas CM, Alves PM, Moreira JL, Carrondo MJ (2000). Effects of ammonia and lactate on growth, metabolism, and productivity of BHK cells. Enzyme Microb Technol.

[16] Giorgi C, Baldassari F, Bononi A (2012). Mitochondrial Ca(2+) and apoptosis. Cell Calcium.

[17] Liu D, He H, Yin D (2013). Mechanism of chronic dietary iron overload-induced liver damage in mice. Mol Med Rep.

[18] Tsien RY (1980). New calcium indicators and buffers with high selectivity against magnesium and protons: design, synthesis, and properties of prototype structures. Biochemistry.

[19] Grimm S, Brdiczka D (2007). The permeability transition pore in cell death. Apoptosis.

[20] Borutaite V, Morkuniene R, Arandarcikaite O, Jekabsone A, Barauskaite J, Brown GC (2009). Nitric oxide protects the heart from ischemia-induced apoptosis and mitochondrial damage via protein kinase G mediated blockage of permeability transition and cytochrome c release. J Biomed Sci.

[21] Liu Y, E Q, Zuo J, Tao Y, Liu W (2013). Protective effect of cordyceps polysaccharide on hydrogen peroxide-induced mitochondrial dysfunction in HL-7702 cells. Mol Med Rep.

[22] Li Y, He K, Huang Y (2010). Betulin induces mitochondrial cytochrome c release associated apoptosis in human cancer cells. Mol Carcinog.

[23] Karl A, Wurm A, Pannicke T (2011). Synergistic action of hypoosmolarity and glutamine in inducing acute swelling of retinal glial (Müller) cells. Glia.

[24] Ohara K, Aoyama M, Fujita M, Sobue K, Asai K (2009). Prolonged exposure to ammonia increases extracellular glutamate in cultured rat astrocytes. Neurosci Lett.

[25] Boulares AH, Yakovlev AG, Ivanova V (1999). Role of poly(ADP-ribose) polymerase (PARP) cleavage in apoptosis. Caspase 3-resistant PARP mutant increases rates of apoptosis in transfected cells. J Biol Chem.

[26] Choi HS, Seo HS, Kim SR (2014). Anti-inflammatory and anti-proliferative effects of Rhus verniciflua stokes in RAW264.7. cells Mol Med Rep.

[27] Bressenot A, Marchal S, Bezdetnaya L, Garrier J, Garrier f, Plénat F (2009). Assessment of apoptosis by immunohistochemistry to active caspase-3, active caspase-7, or cleaved PARP in monolayer cells and spheroid and subcutaneous xenografts of human carcinoma. J Histochem Cytochem.

[28] Gaspers LD, Mémin E, Thomas AP (2012). Calcium-dependent physiologic and pathologic stimulus-metabolic response coupling in hepatocytes. Cell Calcium.

[29] Wang HG, Pathan N, Ethell IM (1999). Ca^2+^-induced apoptosis through calcineurin dephosphorylation of BAD. Science.

[30] Sánchez-Gómez MV, Alberdi E, Ibarretxe G, Torre I, Matute C (2003). Caspase-dependent and caspase- independent oligodendrocyte death mediated by AMPA and kainate receptors. J Neurosci.

[31] Liu Y, Templeton DM (2007). Cadmium activates CaMK-II and initiates CaMK-II-dependent apoptosis in mesangial cells. FEBS Lett.

